# Quantitative risk assessment of radiocesium associated with Japanese foods imported into the United Kingdom

**DOI:** 10.1111/risa.17643

**Published:** 2024-09-12

**Authors:** Amie Adkin, Kay Rylands, Jessica Goodman, Wayne Oatway, Frederique M. Uy, Joanne Edge, Claire Potter

**Affiliations:** ^1^ Food Standards Agency London UK; ^2^ UK Health Security Agency London UK

**Keywords:** food, Fukushima, import controls, quantitative risk assessment, radiological dose assessment

## Abstract

Damage to a nuclear power station resulted in radioactive contamination of certain areas of Japan in 2011. Legislation was put in place in Europe to establish controls on the import of certain types of food and feed, including a limit of 100 radioactive decays (becquerel, Bq) per second of radiocesium per kg. This legislation was retained in the United Kingdom after leaving the EU and then reviewed in 2021. A quantitative risk assessment was developed to estimate the radiological risk to public health from consuming Japanese food imported into the United Kingdom should the maximum level on radiocesium be removed. Although Japanese monitoring data indicated occurrences when products exceeded the 100 Bq per kg limit, these were found to be rare; a total of 1485 occurrences (0.0013%) of all measured foodstuff samples (>1 million) within the scope of this assessment had radiocesium activity concentrations that exceeded 100 Bq per kg. Using the recorded occurrence and level of radiocesium measured, and the current pattern and volume of food imported from Japan, there was an estimated excess risk of fatal cancer of around one in a million per year, categorized as negligible compared to the baseline 2018–2020 UK cancer fatality rate of around 1 in 4. On the basis of the described assessment and the estimated small additional risk, Great Britain lifted import controls related to radioactivity present in food from Japan. A number of recommendations to address data gaps and approaches in this assessment are made, particularly how we can improve modeling UK dietary habits for specialist foods.

## 1 INTRODUCTION

On March 11, 2011, the Great East Japan Earthquake triggered a tsunami that caused an accident at the Fukushima Daiichi nuclear power plant in Japan. A loss of electrical power resulted in a large release of several activation and fission radionuclides: for example, cesium (Cs‐134 and Cs‐137), iodine (I‐131 and I‐129), strontium (Sr‐90), tritium, and plutonium (Pu‐239 and Pu‐240) (United Nations Scientific Committee on the Effects of Atomic Radiation [UNSCEAR], 2020). These were released as direct aquatic discharges and atmospheric releases that were deposited on land and in the Pacific Ocean. These radionuclides contaminated food and fishery products from affected agricultural areas within Japan and the surrounding marine environment.

Immediately following the accident, and to protect people from consuming potentially highly radiologically contaminated foods, restrictions were put in place by the Japanese authorities to both prevent the marketing of the more contaminated foods in Japan and their export to the wider world. In the United Kingdom, the conditions of restricting import of foods from Japan have followed the Commission Implementing Regulation (EU) regulation 2016/6 (The European Commission, 2016). This regulation meant that food produced in Japan could not be imported into the United Kingdom if it had a radiocesium activity concentration exceeding 100 radioactive decays per second (becquerel, Bq) per kg.

It has been more than 10 years since the Fukushima accident, and most radioactive contamination has since decayed from natural processes. However, there remains a potential health hazard from radionuclides with long physical half‐lives, specifically Cs‐137 with a half‐life of 30.1 years and Cs‐134 with a half‐life of 2.1 years. For radiocesium and its progeny, nuclear decay transformations result in emission of ionizing radiation in the form of high‐energy photons (γ‐rays) and β particles. This radiation can damage tissue by causing ionizing events and the production of free radicals, which can in turn result in breakages of chemical bonds in biomolecules such as DNA. γ Photons emitted with the decay of radiocesium are detected using readily available γ‐ray monitors and represent a measure of the chief hazards in this risk assessment.

At very high doses, radiation can kill cells, resulting in the individual showing clinical signs that they had been exposed to radiation in days or weeks. Cell death in significant quantities can result in overt damage to organs and tissues. Such acute tissue reactions will only occur when the dose received exceeds a threshold level whose value depends on which tissue or organ is exposed. The magnitude of damage caused from these deterministic health effects increase with the dose received once the threshold dose is exceeded (International Commission on Radiological Protection [ICRP], 2012). As these effects only occur at high doses, they will not arise as a result of consuming food from Japan and are not considered further in the assessment.

Where doses from exposure to radiation are below the threshold for tissue damage, the main impact on health is the potential for the development of cancer. Within the linear no‐threshold (LNT) model from the ICRP (ICRP, 2005), the internationally accepted approach to practically manage stochastic risks to health, the probability that cancer develops depends on the magnitude of the dose; a higher dose means a greater risk of cancer. It is the probability of these stochastic effects occurring, expressed in terms of either a committed effective dose (CED) or risk of developing cancer, as a result of eating contaminated Japanese imported food for 1 year, which are considered in this risk assessment.

An approximate estimate of the risk from radiation exposure can be obtained using the ICRP (ICRP, 2007) value of 5% per sievert (a measure of the absorbed dose by organs and tissues) for fatal cancer, which represents an average over all ages and both sexes, as well as applying to a globally averaged population. Using this value, an annual CED of 0.02 millisieverts (mSv) corresponds to a risk of fatal cancer of about one in a million, although it should be recognized that the numerical values of risk at such low doses are uncertain.

To quantify the radiological risk to public health from consuming Japanese food imported into the United Kingdom, a quantitative risk assessment was developed to estimate the CED if the maximum level of 100 Bq per kg of radiocesium was removed. In this assessment, the risk of excess cancers was assessed by comparing the estimated CED with a CED of 0.02 mSv per year, which corresponds to an increased risk of cancer of one in a million per year.

## 2 OVERVIEW OF RISK ASSESSMENT

The aim of the risk assessment was to calculate the CED and associated risk to the UK population based on the probability of consuming foods imported from Japan using a number of available data sets.

### 2.1 Activity concentration data

Activity concentrations of combined radiocesium (Cs‐134 and Cs‐137), measured in 1252,017 samples of foods from Japan for the years 2013–2020, were used. These data were then sorted into 30 food groups (Table A1).

### 2.2 Age groups

The assessment was stratified into different UK consumer age intervals to take into account both the various consumption rates of different age groups and also the ICRP (ICRP, 2013) dose coefficients. The age groups considered were Infant (age 4 to less than 18 months), Child 1 (age 18 months to less than 5 years), Child 2 (age 5 to less than 10 years), Child 3 (10 to less than 16 years), Adult (age 16 to less than 70 years), and Women of child‐bearing age (age 16 to less than 50 years).

### 2.3 Implementation of the risk assessment

The risk assessment was developed using @risk version 7.6 (Palisade, 2021), which is a Microsoft Excel–based add‐on Monte Carlo simulation program. The dose estimation was dependent on the following available data:

- Activity concentrations of radiocesium (Bq per kg) measured in Japan in groups of foods.
- Consumption rates (kg per year) for each of the UK population groups for each food group.
- Weight (kg) of each food group imported to the United Kingdom from Japan.

A probabilistic assessment, based on Monte Carlo sampling using distributions reflecting uncertainty or variability, was then developed.

#### 2.3.1 Model overview

**FIGURE 1:**
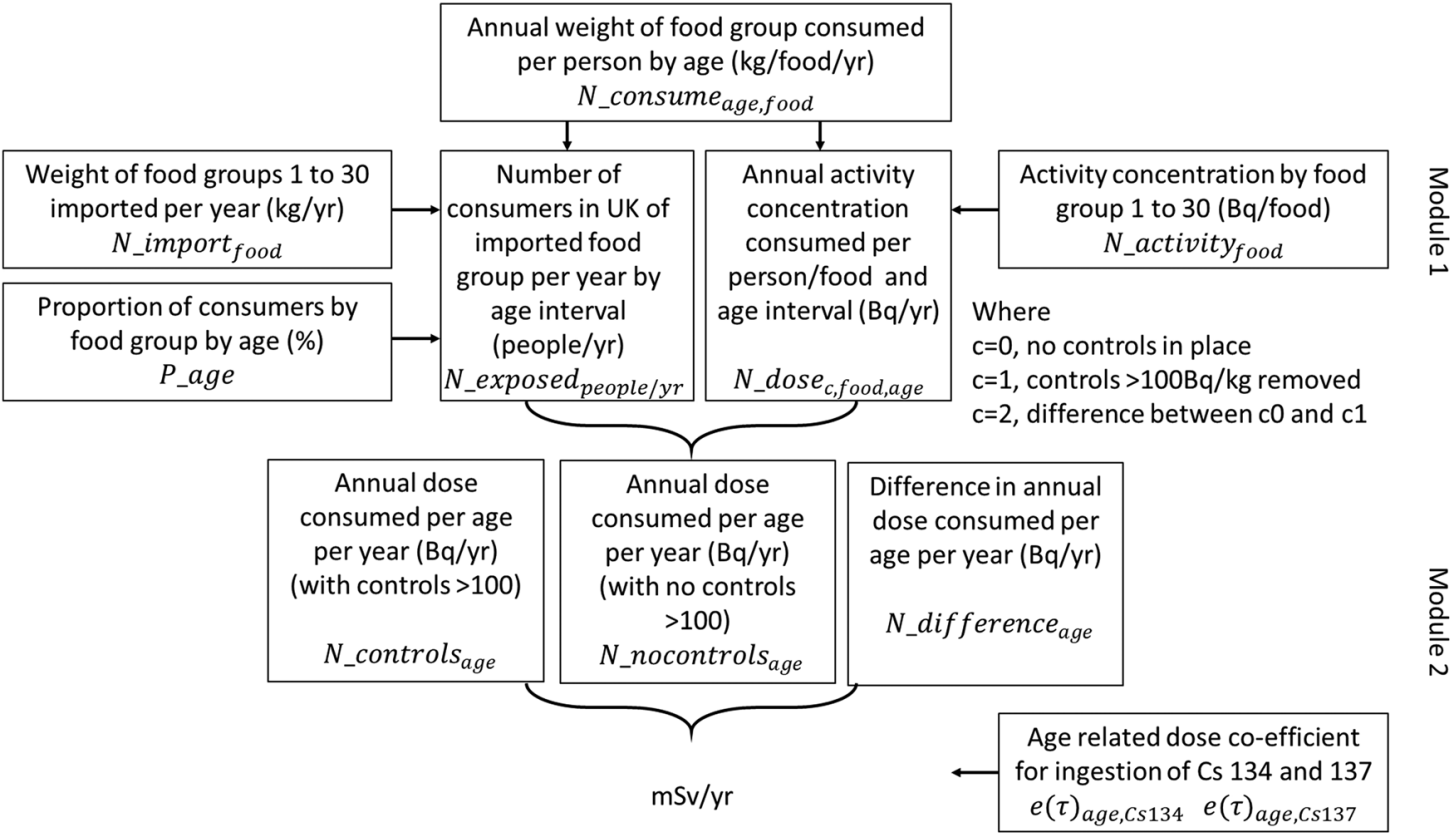
Modular approach to risk pathway.

Risk assessment outputs:

- The annual distribution of CED, for the UK population, by consumption of foods imported from Japan assuming the absence or presence of a 100 Bq per kg level denoted by C = 0, C = 1, respectively, in Figure 1.
- The difference in CED between the distributions in the absence or presence of the 100 Bq per kg level (C = 2 in Figure 1).

The risk assessment was developed as two separate modules (Modules 1 and 2), where the outputs of Module 1 (mean numbers of consumers per food group and annual activity of that food group consumed per person) were used in Module 2 as shown in Figure 1, with the calculations used for each stage described in the following sections.

#### 2.3.2 Parameterization of the model

##### 2.3.2.1 Estimating the number of consumers in the United Kingdom for each imported food group, ($N\_exposed_{people/yr}$)

Import data to the United Kingdom were not available from specific Japanese prefectures. Therefore, whole country import data were available, which contributes to the assessment being conservative. Data on the total weight of each food group imported into the UK from Japan were available and extracted from UK trade information (Her Majesty's Revenue and Customs [HMRC], 2021). This weight of imported foods that could be consumed was based on the finite import patterns of each food group and the UK individual consumption patterns derived from the National Diet and Nutrition Survey (Bates et al., 2014, 2016; Roberts et al., 2018), henceforth referred to as NDNS data, and the Diet and Nutrition Survey of Infants and Young Children (DNSIYC) compiled by the Department of Health (DH, 2013).

For each food group, from 1 to 30, the number of consumers exposed to an imported food group, by age interval ($N\_exposed_{food,age}$), is dependent on the total weight of each food group imported per year ($N\_import_{food}$), the annual weight of consumption of food group by an individual consumer by age interval ($N\_consume_{food,age}$), and the proportion of consumers of imported food group by age interval (P_age).

The estimated weight of any imported food group from Japan for future years was uncertain and described with a Pert distribution using the minimum, most likely^1^ and maximum weight of imported food, for each food group in recent years assuming that import patterns remain stable (further details in Table A1). The Pert distribution—a special case of the β distribution—is nonlinear distribution commonly used in risk analysis to represent uncertainty (Benke & Hamilton, 2008; Benke et al., 2011; Vose, 2008):

$$N\_import_{food}∼Pert\left(minimum,\;most\;likely,\;maximum\right).$$

When estimating how many people in the United Kingdom could consume those imports (as weight of consumption is dependent on age), with no further information, the default assumption was that the proportions of consumers for each age group were the same as the proportion of that age in the total UK population (Table A2) using point values. For some food groups, an exception was made if there was a specific use of a food restricting the age interval of consumers, for example, with alcohol or baby food. In that instance, the distribution would be limited to the age groups that would be assumed to consume food from that group and the ratio of the proportions of the age groups was recalculated.

Due to the high weight of imported food and therefore the considerable number of potential individual consumers across the United Kingdom for most food groups, the model was run (for all food groups) using a sample of 5000 consumers selected at random from across the different age groups. For each consumer, their random age (Age) was estimated by the following equation:

$$Age\;∼\;Discrete\left(\left\{a\right\},\left\{P_{age}\right\}\right),$$

where a is the age group, and P_age is the proportion of that age group in the total population (Office for National Statistics [ONS], 2019). The random weight of the food consumed, for an individual consumer, for that food group, from each age group ($N\_consume_{food,age}$) (kg per year) was then selected from the variable UK consumption distribution based on the NDNS and DH (2013) data as provided in Table A3. The total weight consumed by those randomly selected 5000 consumers was then summed, as estimated by

$$\;{N_{exposed5000}}_{food}=\sum_{1}^{5000}{N_{consume}}_{food}.$$

The total average (mean) number of consumers exposed through a specific food group was then calculated by the following equation:

$$N\_exposed_{food}=\frac{5000}{N\_exposed5000_{food}/N\_import_{food}}.$$

##### 2.3.2.2 Estimating the annual activity concentration consumed per food group per person by age interval, $(N\_dose_{C,food,age}$)

The annual activity consumed was based on the weight of imported food consumed (on a food group basis) per person (on an age group basis) per year.

There was variability associated with both the weight consumed between and within different ages of consumers and the activity concentration of different 1 kg samples of food groups. Therefore, for each individual consuming a particular food, an activity concentration was randomly selected from the distribution for that food and multiplied by the weight of consumption for consumption up to 1 kg, which was randomly selected from the consumption distribution for that age group, $N\_consume_{food,age}$ (kg per year). It was assumed that the activity concentrations were homogeneously distributed within all foods.

When the random annual consumption exceeded 1 kg, the value for the number of kg consumed was rounded to the nearest kg (whole value), and an activity concentration was randomly selected for each kg consumed. This was to reflect the variability of activity per kg in that food group. For each iteration, an activity concentration was randomly selected for each kg up to the maximum number of kgs per year of that food group available for consumption ($N\_dose_{C,food,age}$) as shown in the following equation:

$$\;N\_dose_{C,food,age}=\sum_{N\_consume_{food,age}=1\mathrm{kg}}^{N\_consume_{food,age=\max\mathrm{kg}}}N\_activity_{food},$$

where$\;N\_activity_{food}$ is defined as the activity concentration by food groups 1–30 (Bq per year).

The annual activity consumed per food group was estimated for two different control regimes:

- Where C = 0: No controls on activity concentration were in place. There was no restriction on possible values selected from the distribution of activity concentrations.
- Where C = 1: When a sample selected from the distribution was greater than 100 Bq per kg, it was eliminated and replaced with a resampled value. Therefore, no samples were selected that exceeded 100 Bq per kg.
- The difference in the annual activity concentrations consumed by food group, $N\_dose_{2,food,age}$, was estimated from the difference in activity concentrations between no controls and where controls were in place:
$$\;N\_dose_{2,food,age}=N\_dose_{0,food,age}\;-N\_dose_{1,food,age}.$$

##### 2.3.2.3 Estimating the annual activity ingested by exposed consumers with ($N\_controls_{age}$) and without ($N\_nocontrols_{age}$) controls in place (Bq per year)

Using the individual food group results from Module 1, Module 2 was used to calculate the distribution of ingested activity, and hence dose, from the consumption of these food groups per age group. In the next step, the dose from the total diet was determined when the 100 Bq per kg control was present or absent, and calculated the difference between these two doses.

The consumption of the food groups was assessed in a tiered approach. The first tier consumed all 30 food groups from Japan representing the highest consumers. However, the quantity of “eggs” imported from Japan was small, and all Japanese eggs were consumed by this first tier. This means the second tier of consumers would only consume 29 food groups, until the next food group (imported “infant formula”) was also exhausted. The third tier of consumers consumed 28 food groups, and so on, with the final tier consuming “condiments, spices, and preserves” (includes soy sauce). Note that for all age groups other than adults, the amount of alcohol consumed per year is 0 kg. Similarly, “infant formula” is only consumed by infants, and “baby food” is only consumed by the “Infants,” “Child 1,” and “Child 2” age groups (refer to Table A3 for a list of dietary exclusions).

To implement the tiered approach, the radiological dose that any individual food group can contribute to personal dietary exposure is assumed to be $N\_dose_{C,i,\;age\;}$, where *i* is the *i*th food group, that is, *i* = 1, 2, 3, … 30, *C* is the control regime, and *age* is the age group consuming the *i*th food group as described in Section 2.3.1. The uncertainty associated with $N\_dose_{C,i,\;age\;},$ as estimated by simulation in Module 1, was described in Module 2 with a cumulative distribution, using the minimum, maximum, and 5th and 95th percentile measures.

As imported volumes for each food group are finite, there is an upper limit on the number of individuals that can consume that food in a year. These upper limits on the number of consumers are denoted by $N\_exposed_{food,age,i}$ (*i* = 1…30). For simplicity, we have ordered the foods so that $N\_exposed_{food,age,1}$ < $N\_exposed_{food,age,2}$ …. < $N\_exposed_{food,age,30}$. It is also helpful to conceive of a notional $N\_exposed_{food,age,0}$ such that $N\_exposed_{food,age,0}$ = 0.

With these definitions in place, it follows that the overall radiological exposure of an individual consumer, for C = 0, with no controls in place is given by

$$N_{\mathrm{nocontrol}s_{\mathrm{age}}}=\sum_{i=1}^{30}N\_dose_{0,age,i}\;.$$

In addition, where C = 1, where a selection from the distribution was greater than 100 Bq per kg:

$$N_{\mathrm{control}s_{\mathrm{age}}}=\sum_{i=1}^{30}N\_dose_{1,age,i}.$$

In addition, where C = 2, estimating the difference between no controls and controls:

$$N_{\mathrm{differenc}e_{\mathrm{age}}}=\sum_{i=1}^{30}N\_dose_{2,age,i}\;.$$

There is a maximum of $N\_exposed_{food,age,1}$ individuals in the population who fall within this top tier of exposure. The second tier of overall radioactive exposure is at a level of

$$N_{\mathrm{nocontrol}s_{\mathrm{age}}}=\sum_{i=2}^{30}N\_dose_{0,age,i},$$

$$N_{\mathrm{control}s_{\mathrm{age}}}=\sum_{i=2}^{30}N\_dose_{1,age,i}\;,$$

$$N_{\mathrm{differenc}e_{\mathrm{age}}}=\sum_{i=2}^{30}N\_dose_{2,age,i}\;,$$

and there is a maximum of ($N\_exposed_{food,age,2}$–$N\_exposed_{food,age,1}$) individuals within the second tier. This can be summarized in a single equation covering all 30 tiers of exposure, which is as follows. The exposure at the *j*th tier is

$$N_{\mathrm{nocontrol}s_{\mathrm{age}}}=\sum_{i=j}^{30}N\_dose_{0,age,i}\;,$$

$$N_{\mathrm{control}s_{\mathrm{age}}}=\sum_{i=j}^{30}N\_dose_{1,age,i}\;,$$

$$N_{\mathrm{differenc}e_{\mathrm{age}}}=\sum_{i=j}^{30}N\_dose_{2,age,i},$$

where *j* = 1 denotes the highest tier of potential radiation exposure from the diet, and where *j* = 30 denotes the lowest tier.

The number of consumers within each of the tiers is ($N\_exposed_{food,age,j}$‐ $N\_exposed_{food,age,j-1}$), where $N\_exposed_{food,age,}$_0_ = 0.

The total number of consumers within any of these tiers of exposure is $N\_exposed_{food,age,}$_30_. Under this pessimistic assumption, the weights of all imported products are consumed, and all exposure to Fukushima radiocesium is concentrated within a subset of the UK population with all other members of the population having zero exposure.

A discrete function was used to randomly select the tier exposure for that iteration, where the probability of selecting a tier was proportional to the number of individuals exposed, $\;N\_exposed_{food,age}$. For example, due to the number of available imports, there are far fewer individuals who can consume all imported products as part of their total diet, and this was therefore far less likely to be selected. The most commonly selected individual was one who consumed condiments, sauces, and preserves only. The resulting distribution represents the total uncertainty associated with the annual consumption of an individual UK consumer of a diet including Japanese imported foods in the United Kingdom.

##### 2.3.2.4 Estimated committed effective dose (CED) (mSv per year) from the activity ingested

The unit output of Bq per year ingested was converted to CED in units of mSv per year using the following equation. The ratio of Cs‐137 to Cs‐134 was assumed to be 1:1 to reflect the pattern seen in the data. This is known to be a cautious approach and likely to overestimate the amount of Cs‐134 (refer to Section 5.2). For example, the annual CED from the diet, consumed with no controls, would be calculated by

$$\begin{array}{ccc} \mathrm{CED}\; & = & \;e{\left(\tau \right)}_{\mathrm{age},\;\mathrm{Cs}137}\;\times \left[\frac{N\_\mathrm{nocontrol}s_{\mathrm{age}}}{2}\right]\\ & & +\;e{\left(\tau \right)}_{\mathrm{age},\;\mathrm{Cs}134}\;\times \left[\frac{N\_\mathrm{nocontrol}s_{\mathrm{age}}}{2}\right], \end{array}$$

where $e{\left(\tau \right)}_{\mathrm{age},Cs-137}$ is the age‐related dose coefficient for ingestion of *C*s‐137 and $e{\left(\tau \right)}_{\mathrm{age},\mathrm{Cs}134}$ is the age‐related dose coefficient for ingestion of Cs‐134 (units of Sv per Bq).

The age‐related dose coefficients are discussed in Section 2.3.2.9. The above equation was used for dose conversions from Bq per year to mSv per year for formulae producing outputs in activity ingested (i.e., $N\_difference_{age}$, $N\_controls_{age}$).

##### 2.3.2.5 Weight of food groups 1–30 imported per year, $N\_import_{food}$ (kg per year)

Annual import data from Japan to the United Kingdom (kg per year) were obtained for 5 years (HMRC, 2021) and attributed to the 30 food groups under consideration. The annual weight for each food group varies in each year and presents some uncertainty about future imported levels. This uncertainty was described using a pert distribution using the minimum and maximum values from the import data and generating the most likely value such that the distribution mean equaled the observed import mean weight (Table A1). For most food groups, the most recent 5 years of data was used to estimate the minimum, mean, and maximum imported values. However, in food groups where fewer data were available, or higher variability between years was observed, more than 5 years of import data were used (Table A1).

##### 2.3.2.6 Annual weight of consumption of food groups 1–30 by one person by age, $N\_consume_{age,\;food}$ (kg per year)

Consumption data for the UK population were based on NDNS and DH (2013) data. Distributions of the variable consumption of each food group in the United Kingdom, by age interval, were calculated. The known variability was described using a log‐normal distribution, based upon observed/recorded consumption data values (Table A2) and determined by Akaike's information criterion (AIC) in @risk, an index that evaluates how well a model fits to the data. Where there was limited or no data available on certain food group/age interval combination, assumptions were made (Table A3). For example, there are no specific data on the annual consumption distributions for those consumers over 70 years old. The assumption was made that this group consumed the same amounts as other adults.

##### 2.3.2.7 Proportion of consumers of imported food group by age interval, P_age (%)

The age of UK consumers eating Japanese imported foods is not known, and with no further data, an assumption was made that the age proportions of consumers were the same as the proportion of that age in the total UK population using point values shown in Table A4.

##### 2.3.2.8 Activity concentration by food group 1 to 30, $N\_activity_{food}$ (Bq per year)

The activity concentration data from the Japanese Government (Ministry of Health, Labour and Workforce [MHLW], 2021) varied considerably both between food groups and within food groups sampled. This variability has been incorporated into the assessment using a best fitting distribution based on the mean and the maximum values. Best fitting distributions were first ranked by AIC. A maximum value 25% higher than the observed maximum value was then used as the cut‐off point. This value was based on the assumed detection efficiencies of the instruments used and the sampling of the tested foods in Japan. Distributions for the sampled activity concentrations for each commodity are shown in Table A5.

##### 2.3.2.9 Age‐related dose coefficient for ingestion of Cs‐137 and Cs‐134, $e{\left(\tau \right)}_{age,Cs137}$ and $\;e{\left(\tau \right)}_{age,Cs134}$

To convert the calculated radiocesium activity ingested per food group and age, the value is multiplied by the age‐related dose coefficient for ingestion. The dose coefficients for ingestion of Cs‐137 and Cs‐134 for each age group are shown in Table A4 (ICRP, 2020).

## 3 SENSITIVITY ANALYSES

A multivariate stepwise regression analysis was used to calculate linear regression or sensitivity values for each input parameter represented by a distribution for Module 2 outputs of the annual distribution of activity concentration. This approach is preferred for large numbers of input parameters, as all variables that provide an insignificant contribution are removed from the analysis (Frey & Patil, 2002).

## 4 RESULTS

Results are shown in Table 1 and Table A7 of the Supporting Information section. Both variability and uncertainty are considered in the assessment and are represented by 5th and 95th percentiles (within parentheses), which indicate the range within which 90% of the results lie. The greater the range between the percentiles, the greater the total uncertainty. Module 1 was run for 50,000 iterations using Latin Hypercube sampling to reach convergence. Module 2 converged at approximately 400,000 iterations.

**TABLE 1:** Estimated annual dose (millisieverts [mSv] per year) for a representative UK consumer by age (dose conversion from Bq per kg to committed effective dose [CED]).

Age	$N\_nocontrols_{age}$ Mean (5th, 95th) (mSv/year)	$N\_controls_{age}$ Mean (5th, 95th) (mSv/year)	$N\_difference_{age}$ Mean (5th, 95th) (mSv/year)
Infant (4–18 months)	4.8 × 10^−4^	4.8 × 10^−4^	2 × 10^−6^
	(7 × 10^−6^, 1.9 × 10^−3^)	(7 × 10^−6^, 1.9 × 10^−3^)	(0, 5 × 10^−20^)
Child 1 (18 months to <5 years)	8.1 × 10^−4^	8.1 × 10^−4^	2.1 × 10^−6^
	(2.2 × 10^−5^, 3.8 × 10^−3^)	(2.2 × 10^−5^, 3.8 × 10^−3^)	(0, 9.9 × 10^−20^)
Child 2 (5 to <10 years)	8.3 × 10^−4^	8.4 × 10^−4^	2.2 × 10^−6^
	(2.8 × 10^−5^, 4 × 10^−3^)	(2.8 × 10^−5^, 4 × 10^−3^)	(0, 8.5 × 10^−20^)
Child 3 (10 to <16 years)	1.2 × 10^−3^	1.2 × 10^−3^	6.7 × 10^−6^
	(2.7 × 10^−5^, 6.3 × 10^−3^)	(2.7 × 10^−5^, 6.3 × 10^−3^)	(0, 1.1 × 10^−19^)
Adults (16–<70 years) excluding females (16–<50 years)	1.6 × 10^−3^	1.6 × 10^−3^	8 × 10^−6^
	(3.5 × 10^−5^, 7.2 × 10^−3^)	(3.5 × 10^−5^, 7.2 × 10^−3^)	(0, 1.9 × 10^−19^)
Female (16–<50 years)	1.5 × 10^−3^	1.5 × 10^−3^	6.9 × 10^−6^
	(2.9 × 10^−5^, 7 × 10^−3^)	(2.9 × 10^−5^, 7 × 10^−3^)	(0, 1.4 × 10^−19^)
(Fetus) Female (16–<50 years)	6.7 × 10^−4^	6.6 × 10^−4^	3.1 × 10^−6^
	(1.3 × 10^−5^, 3.2 × 10^−3^)	(1.3 × 10^−5^, 3.2 × 10^−3^)	(0, 6.1 × 10^−20^)

The annual activity consumed per person by age interval, $N\_dose_{c,food,age}$, was estimated for each of the 30 food groups based on the amount consumed and activity concentrations estimated from monitoring results. The highest estimated mean activity ingested for an adult in the absence of controls was 1457 Bq per year from the nonleguminous green vegetable (NLGV) food group. Using conservative assumptions to combine the individual food groups together into a person's annual diet, an estimated average of 2197,869 people could consume some type of Japanese imported food per year. The top three products consumed were condiments (including soy sauce), shoots, and ready‐to‐eat foods. Of adult consumers, an estimated mean (5th–95th percentiles) activity of 100.6 (2.2–452.1) Bq per year (equating to a CED of 1.6 × 10^−3^ mSv per year) was found to be consumed in the absence of controls and 99.8 (2.2–452) Bq per year (equating to a CED of 1.6 × 10^−3^ mSv per year) consumed when the controls are in place.

The differences between the calculated doses in the presence and absence of controls for a representative consumer have been estimated separately. The distribution is extremely skewed with the majority of values being 0, that is, no difference for that consumer, with very infrequent higher values when a food serving is consumed that would have been restricted with controls in place. The mean difference between the presence and absence of the controls for an adult consumer is 0.50 (0, 1 × 10^−15^) Bq per year which equates to a CED of 8 × 10^−6^ mSv per year. Results for all age groups are provided in Table A7 as activity ingested, and Table 1 shows the results converted to CED in mSv per year.

The contribution by food group to the difference in annual dietary activity (i.e., dose in the absence of controls minus the dose in the presence of controls) was estimated. The top three contributors to adult dietary dose were NLGV (27%), saltwater fish (16%), and rice (16%). Results were slightly different for infants due to differing consumption rates, for which group the top three were fruit (16%), NLGV (13%), and saltwater fish (11%).

### 4.1 Sensitivity analysis results

For Module 2, the top two significant inputs were ranked by regression coefficient for the annual distribution of activity concentration under no controls, controls applied, and the difference in controls as shown in Table 2. The most significant uncertainty for the distribution of total diet activity ingested was from the uncertainty associated with the activity concentration of condiments, sauces, and preserves, which includes soy sauce. For the difference between control measures, $N\_difference_{age},$ the uncertainty associated with food groups that have been monitored in Japan with values above 100 Bq per kg was significant, including NLGV and saltwater fish for adults, and cereals and rice for infants and young children (Child 1).

**TABLE 2:** Significant contributing inputs to annual activity ingested.

Age	$N\_nocontrols_{age}$	$N\_controls_{age}$	$N\_difference_{age}$
Infant (4–18 months)	CSP, RTE	CSP, RTE	NLGV, cereals
Child 1 (18 months to <5 years)	CSP, rice	CSP, NLGV	NLGV, rice
Child 2 (5–<10 years)	CSP, RTE	CSP, RTE	NLGV, saltwater fish
Child 3 (10–<16 years)	CSP, root vegetables	CSP, RTE	NLGV, saltwater fish
Adults (16–<70 years) excluding females (16–<50 years)	CSP, RTE	CSP, RTE	NLGV, saltwater fish
Female (16–<50 years)	CSP, RTE	CSP, RTE	NLGV, saltwater fish

Abbreviations: CSP, condiments, sauces, and preserves (includes soy sauce); NLGV, nonleguminous green vegetables; RTE, ready‐to‐eat foods.

## 5 DISCUSSION

The probabilistic assessment using the weight of imported foods and annual UK consumption rates estimates the number of consumers affected as approximately 2.2 million and initially seems high when considering the total UK population size of approximately 67 million. However, among the food items imported are condiments, including imported soy sauce, which are consumed widely and in small serving sizes.

Uncertainties associated with the NLGV food group had the greatest impact on results when comparing the difference made by implementing controls. NLGV are shown to have the highest impact on estimated annual activity ingested per adult of the 30 food groups and contributed the most to the overall dietary activity ingested. This is because samples from the NLGV food group include measurements of Koshiabura, a specialty wild plant with recorded activity concentrations of up to 12,000 Bq per kg total radiocesium. By including these samples with high activity concentrations, the assessment predicted a rare but possible occurrence of such levels of radioactivity in some imported servings of NLGV. The results were therefore skewed toward a probability distribution with very occasional but very high values at the tail end of the distribution. However, as these samples were from 2013, results may represent a cautious overestimation of dose within this food group; presently, Koshiabura and other wild plants are not a commodity permitted for import but were included in this assessment to give a cautious estimate of possible high doses from green vegetables.

The uncertainty of the saltwater fish food group was identified as the second greatest impact on the results and had the second highest food group activity ingested per year for an adult and the second highest food group contributor to the overall dietary activity ingested.

The probabilistic assessment included some rare but high values of activity concentrations of up to a 25% increase in the monitored values. This was to compensate for the fact that not all foods had been tested, and that the maximum values might have been missed by the sampling method. Although higher maximum values were rare, they resulted in the mean activity concentrations being increased.

A deterministic calculation was also carried out for the assessment of risk to the UK population from lifting controls that were initially imposed as a result of potential radioactivity in Japanese foodstuffs and is documented and discussed in Food Standards Agency (FSA) (2021) and references therein.

### 5.1 Uncertainty and variability

Table A1 shows various input parameters, and which of those are associated with quantified variability and uncertainty. Calculated values such as annual distributions will not add to the uncertainty and variability, but the source data used in the model, such as activity concentrations measured in the food groups, consumption rates for each of the UK population groups for each food group, and weight of imports, will contain variability and may also contribute to uncertainties if there are data gaps (see Section 5.2). The inherent variability in the radiocesium data, import data, and consumption data is taken into account by using the range and spread of probability distributions.

There is also uncertainty around the limit of detection (LOD), a level of radioactivity below which radioactive events are undetectable with the monitoring instruments used. The LOD value was used to quantify all the monitoring data below the LOD. Overall, 90% of samples were below the LOD but above zero, leading to a systematic bias on average for radiation levels above or equal to the LOD value.

The use of total radiocesium activity concentration in this model, and assuming a 1:1 Cs‐134 to Cs‐137 ratio, has added to the uncertainty and probably overestimated the CED as it is unlikely that Cs‐134 and Cs‐137 will be present in equal amounts because Cs‐134 decays much faster than Cs‐137 (Leggett, 2013).

Other factors adding to uncertainties are aggregation errors, such as the grouping method used to categorize the various food groups and matching the population age groups to the available consumption data and ICRP (2013) reference ages. Rounding consumption values of less than 1 kg to whole numbers will lead to uncertainty by overestimating the CED. There is also uncertainty in the way professional judgment was used to decide on classification of certain food types into a food group and inherent uncertainty in consumption data (FSA, 2021).

### 5.2 Key assumptions and data gaps

Several assumptions were made during the risk assessment process that need to be highlighted when interpreting the results. These are listed as follows:

- It was assumed that consumers would source their diet from Japanese imports where foods were sufficiently available (limited due to the finite weight of food imported). This may be unrealistic and lead to an overestimation of the estimated dose for the highest and lowest consumers using the tiered approach to combine individual food groups. If there were more realistic estimates of the share of the diet from imported food for an individual, and these were on average lower, the imported food would be consumed at a lower level and therefore would lead to an increase in the number exposed. However, the national annual dose imported would remain unchanged.
- It was assumed that the age proportions of consumers for Japanese imported foods were the same as the proportion of the age in the UK population using point values. A richer data set on the complete diet for those consuming any Japanese foods would reduce this uncertainty, which is not quantified in the risk assessment.
- The activity concentrations in food groups from 2013 to 2020 are assumed to be representative of future levels. However, the activity concentrations of radiocesium are likely to reduce in future years due to natural decay processes. The use of data from 2013 to 2020 will therefore overestimate future risk.
- Imported foods from Japan vary from year to year, and the assessment uses previous years’ import data. Results from the assessment could be underestimated or overestimated if the amount of food imported were to increase or decrease significantly in future years or the pattern of imported food groups changes.
- Finally, it is assumed that activity concentrations are distributed uniformly within the food or beverages, whereas the activity distributions may be nonuniform.

Recommendations to address data gaps and approaches in this assessment may take the form of a more realistic measure of Japanese food consumption in the United Kingdom when combined into an entire diet and modeling the variability among consumers. The stochastic exposure assessment method for a combined diet was challenging to develop in this risk assessment for a number of reasons, including a limited source material (annual imports from Japan) and the variability between consumers for such specialist foods. Unlike single‐ingredient risk assessments, where assessors may take the 95th percentile of consumption as a conservative estimate, when combining all the ingredients at the 95th percentile into a diet, these estimates resulted in impossibly high levels of annual consumption, focused on only a few individuals before reaching the finite weight of the food imported into the United Kingdom. The approach taken in this article is simplified—limiting the number of assumptions used—placing the conversative assumption that exposed consumers source their diet from Japanese imports where foods were sufficiently available. We are currently investigating how we can improve our methods as to how other data sources or surveys could be incorporated to more accurately model UK dietary habits for specialist foods and reduce uncertainties.

Another improvement that could be made to the assessment would be associated with the LOD with estimates of monitoring results reported as <LOD rather than simply assuming they are all at the LOD.

However, it is unlikely that the LOD could be improved without a significant technological investment as methods have already been optimized for such low levels of radiation (International Atomic Energy Agency, 2004). Nevertheless, an area that may be more fruitful is the further development of a consensus method for the analyses of LOD data in radiation protection.

## 6 CONCLUSION

A probabilistic risk assessment was conducted to estimate the CED to the representative person (adult) should UK import controls be removed for food imported from the Japanese Fukushima region a decade after the Fukushima radiological accident. That assessment estimated the CED to be around 1.6 × 10^−3^ mSv per year should controls be lifted. The probabilistic estimate of the additional (incremental) dose if restrictions were lifted was calculated to be 8 × 10^−6^ mSv per year and judged to be negligible even when compared to the lower limit of the 1–20 mSv per year ICRP (2007) reference levels for existing exposures. These calculated doses roughly equate to an excess risk of fatal cancer of less than 1 in a million per year, which is negligible compared to the baseline 2018–20 cancer fatality rate of 1 in 4 (Cancer Research UK, 2022). Such exposure would therefore not be expected to cause significant radiological risk to the UK population. On the basis of the described assessment and the estimated small additional risk, Great Britain lifted import controls that were initially imposed as a result of potential radioactivity in Japanese foodstuffs.

## Supporting information

Supporting Information

## Footnotes

1 The “most likely” value was estimated using the mean value of the weight of the imported food and fitting the assumed Pert distribution.

## 1

**TABLE A1:** Variables included in the radiocesium risk assessment with table references to the Supporting Information section.

Description	Symbol	Distribution	Data set	Units	Uncertainty and variability	Reference
Weight of food groups 1–30 imported per year	$N\_import_{food}$	Pert	Table A1	kg/year	Uncertainty around the mean of the distribution	HMRC (2021)
Annual weight of consumption of food groups 1–30 by one person by age interval	$N\_consume_{food,age}$	Lognormal	Table A2	kg/year	Variability in individual consumption and uncertainty due to short sampling window	NDNS (Bates, 2014, 2016)
Proportion of consumers in the United Kingdom by age interval	$P_{age}$	Point value	Table A3	%	N/A	ONS (2019)
Mean number of consumers of imported food group per year	$N\_exposed_{food,age}$	Point value	Calculated value	People/year	N/A	N/A
Activity concentration by food groups 1–30	$N\_activity_{food}$	Fitted	Table A5	Bq/year	Variability in annual mean A/C and uncertainty in LOD	MHLW (2021)
Annual activity concentration consumed of food group per person by age interval	$N\_dose_{C,food,age}$	Lognormal	Calculated value	Bq/year	Uncertainty and variability	N/A
Annual distribution of activity consumed by consumers exposed (with controls >100)	$N\_controls_{age}$	Discrete	Calculated value	Bq/year	Uncertainty and variability	N/A
Annual distribution of activity consumed by consumers exposed (with no controls)	$N\_nocontrols_{age}$	Discrete	Calculated value	Bq/year	Uncertainty and variability	N/A
Difference in the distribution of activity consumed by consumers exposed	$N\_difference_{age}$	Discrete	Calculated value	Bq/year	Uncertainty and variability	N/A
ICRP (2002, 2012) Dose coefficients by age group and fetus and embryo	Dose	Point value	Table A6	mSv/Bq	Variability between Cs‐134 and Cs‐137, mean value used	ICRP (2002, 2012)

## References

[risa17643-bib-0001] Bates, B. , Lennox, A. , Prentice, A. , Bates, C. , Page, P. , Nicholson, S. , & Swan, G. (2014). National diet and nutrition survey results from years 1, 2, 3 and 4 (combined) of the rolling programme (2008/2009–2011/2012) . Public Health England, Food Standards Agency. https://assets.publishing.service.gov.uk/government/uploads/system/uploads/attachment_data/file/594360/NDNS_Y1_to_4_UK_report_executive_summary_revised_February_2017.pdf

[risa17643-bib-0002] Bates, B. , Cox, L. , Nicholson, S. , Page, P. , Prentice, A. , Steer, T. , & Swan, G. (2016). National diet and nutrition survey results from years 5 and 6 (combined) of the rolling programme (2012/2013–2013/2014) . Public Health England, Food Standards Agency. https://assets.publishing.service.gov.uk/government/uploads/system/uploads/attachment_data/file/551352/NDNS_Y5_6_UK_Main_Text.pdf

[risa17643-bib-0003] Benke, K. K. , & Hamilton, A. J. (2008). Quantitative microbial risk assessment: Uncertainty and measures of central tendency for skewed distributions. Stochastic Environmental Research and Risk Assessment, 22(4), 533–539.

[risa17643-bib-0004] Benke, K. K. , Steel, J. L. , & Weiss, J. E. (2011). Risk assessment models for invasive species: Uncertainty in rankings from multi‐criteria analysis. Biological Invasions, 13, 239–253.

[risa17643-bib-0005] Cancer Research UK . (2022). Cancer mortality for all cancers combined . Cancer Research UK. https://www.cancerresearchuk.org/health‐professional/cancer‐statistics/mortality/all‐cancers‐combined

[risa17643-bib-0006] Commission implementing regulation (EU) 2016/6 of 5 January 2016 imposing special conditions governing the import of feed and food originating in or consigned from Japan following the accident at the Fukushima nuclear power station and repealing implementing regulation (EU) No 322/2014. Official Journal of the European Union, L 3/5, 5–15.

[risa17643-bib-0007] Department of Health . (2013). Diet and nutrition survey of infants and young children (DNSIYC), 2011 . Department of Health and Social Care: UK Government.

[risa17643-bib-0009] Food Standards Agency . (2021). Quantitative risk assessment of radiocaesium in Japanese foods. UK government report . Food Standards Agency. https://www.food.gov.uk/evidence/quantitative‐risk‐assessment‐of‐radiocaesium‐in‐japanese‐foods

[risa17643-bib-0010] Frey, C. H. , & Patil, S. R. (2002). Identification and review of sensitivity analysis methods. Risk Analysis, 22(3), 553–578. 12088234

[risa17643-bib-0011] UK Government, Her Majesty's Revenue and Customs [HMRC] Trade data website . (2021). Available at: https://www.uktradeinfo

[risa17643-bib-0012] International Atomic Energy Agency . (2004). Quantifying uncertainty in nucler analytical measurements (Research Report No. IAEA‐TECDOC‐1401). IAEA.

[risa17643-bib-0014] International Commission on Radiological Protection . (2002). Doses to the embryo and fetus from intakes of radionuclides by the mother ICRP publication 88. Annals of the ICRP, 30(1–3), 19. 10.1016/S0146-6453(01)00022-7 11730884

[risa17643-bib-0015] International Commission on Radiological Protection . (2005). Publication 99: Low‐dose extrapolation of radiation‐related cancer risk . ICRP.

[risa17643-bib-0016] International Commission on Radiological Protection . (2007). The 2007 recommendations of the International Commission on Radiological Protection [ICRP Publication 103]. ICRP. 10.1016/j.icrp.2007.10.003 18082557

[risa17643-bib-0017] International Commission on Radiological Protection . (2012). ICRP statement on tissue reactions and early and late effects of radiation in normal tissues and organs—Threshold doses for tissue reactions in a radiation protection context [ICRP Publication 118]. ICRP. 10.1016/j.icrp.2012.02.001 22925378

[risa17643-bib-0018] International Commission on Radiological Protection . (2013). Compendium of Dose Coefficients based on ICRP Publication 60. ICRP Publication 119. Annals of the ICRP, 41(Suppl 1). 10.1016/j.icrp.2012.06.038 23025851

[risa17643-bib-0019] Leggett, R. W. (2013). Biokinetic models for radiocaesium and its progeny. Journal of Radiological Protection, 33, 123–140. 23296405 10.1088/0952-4746/33/1/123

[risa17643-bib-0020] Ministry of Health, Labour and Welfare . (2021). Levels of radioactive materials in foods tested in respective prefectures: Japanese government . Ministry of Health, Labour and Welfare. https://www.mhlw.go.jp/english/topics/2011eq/index_food_radioactive.html https://www.mhlw.go.jp/english/topics/2011eq/dl/food‐130926_1.pdf https://www.maff.go.jp/e/export/pdf/safety_en_210129.pdf

[risa17643-bib-0021] Office for National Statistics . (2019). Population estimates for the UK, England and Wales, Scotland and Northern Ireland: mid‐2019.

[risa17643-bib-0022] Palisade . (2021). @Risk Version 7.6 [Computer program]. https://www.palisade.com/risk/default.asp

[risa17643-bib-0023] Roberts, C. , Steer, T. , Maplethorpe, N. , Cox, L. , Meadows, S. , Page, P. , Nicholson, S. , & Swan, G. (2018). National diet and nutrition survey results from years 7 and 8 (combined) of the rolling programme (2014/2015–2015/2016) . Public Health England, Food Standards Agency. https://assets.publishing.service.gov.uk/government/uploads/system/uploads/attachment_data/file/699241/NDNS_results_years_7_and_8.pdf

[risa17643-bib-0024] United Nations Scientific Committee on the Effects of Atomic Radiation . (2020). Report. SCIENTIFIC ANNEX B: Levels and effects of radiation exposure due to the accident at the Fukushima Daiichi Nuclear Power Station: Implications of information published since the UNSCEAR 2013 report . United Nations.

[risa17643-bib-0025] Vose, D. (2008). Risk analysis: A quantitative guide. John Wiley & Sons.

