# Supplementary material for: Quantitative risk assessment of radiocesium associated with Japanese foods imported into the United Kingdom
Source: Risk Anal. 2024 Sep 12;45(5):1105–14. doi: 10.1111/risa.17643 (PMC12087749; doi:10.1111/risa.17643)
Supplement: Supplementary file 1 — Supporting Information [file RISA-45-1105-s001.docx]

Supplementary online materials

Identification of food groups

The final number of food groups which could be defined was 3045. Of these, 35 groups of food were identified and 30 were parameterised.

The 30 food groups included in the assessment were: alcoholic beverages; baby food; bread; cattle; cereals and grains; confectionary; condiments, sauces and preserves (CSP); dairy products; dried fruit, nuts and seeds (DFNS); dried fish; eggs; fats and oils; freshwater fish; fruit; infant formula; leguminous vegetables; meat and dairy alternatives; mushrooms; non-leguminous green vegetables (NLGV); other meats; pasta; potatoes; ready-to-eat-foods (RTE); rice; root vegetables; saltfish; seafood – other; seaweed and algae; shoots; and soft beverages. Five food groups were removed for the following reasons: no activity concentrations were available in the sample data for ‘Yeast’, there was insufficient sample data to provide distributions required for ‘Dried mushroom’. There was no import data for ‘Algae’ and insufficient import data to draw distributions for ‘Snacks’. The ‘Cultivated mushroom’ food group was combined with ‘Mushroom’ food group to form one ‘Mushroom’ food group.

Table A1: Weight imported for each food group parameter values and distribution used in the risk assessment (kg/year)

| Food group | Distribution and values |
| --- | --- |
| Alcoholic beverages | Pert(397658,502007.95,719877) with a mean of 520927.8 kg/yr based on 5 years of trade data |
| Baby Food | Pert(1380,3584.75,8167) mean 3981 kg/yr based on 5 years trade data |
| Bread | Pert(3872,12042.5,18386) with a mean of 11738 kg/yr based on 5 years of trade data |
| Cattle | Pert(36288,44066,62282) with a mean of 45805 kg/yr based on 6 years of trade data |
| Cereals and grains | Pert(87959,116200,134682) with a mean of 114573.4 kg/yr based on 5 years of trade data |
| Condiments, spices, preserves | Pert(4745848,5174931,5643423) with a mean of 5174931 kg/yr based on 5 years of trade data |
| Confectionary | Pert(15165,76426.8,100863) with a mean of 70289.2 kg/yr based on 5 years of trade data |
| Dairy products | Pert(5,17.75,42) with a mean of 19.67 kg/yr based on 3 years of trade data available |
| Dried fish | 1300 from one year trade data available |
| Dried food, nuts and seeds | Pert(75174,90962.7,102330) with a mean of 90225.8 kg/yr based on 5 years of trade data |
| Eggs | 12 from one year trade data available |
| Fats and oils | Pert(6712,85105.55,237867) with a mean of 97500.2 kg/yr based on 5 years of trade data |
| Freshwater fish | Pert(651,40441.25,220504) with a mean of 63820 kg/yr based on 5 years of trade data |
| Fruit | Pert(1203,5625.5,12427) with a mean of 6022 kg/yr based on 5 years of trade data |
| Infant formula | Pert(1380,3584.75,8167) with a mean of 3981 kg/yr based on 5 years of trade data |
| Leguminous green vegetables | Pert(131,1019.6,5429) with a mean of 1606.4 kg/yr based on 5 years of trade data |
| Meat and dairy Alternatives | Pert(352096, 9725, 43764.83) with a mean 326821.8 kg/yr based on 5 years trade |
| Mushrooms | Pert(96,212,700) mean of 274 kg/yr based on 5 years of trade data |
| NLGV | Pert(174319,213463.1,282519) with a mean 218448.4 kg/yr based on 5 years trade |
| Other meats | Pert(4742,23070.75,43543) with a mean of 23428 kg/yr based on 5 years of trade data |
| Pasta | Pert(390416,480931.05,726139) with a mean of 506713.2 kg/yr based on 5 years of trade data |
| Potatoes | Pert(150,711.85,2333) with a mean of 888.4 kg/yr based on 5 years of trade data |
| RTE foods | Pert(1972306,2247623.55,2450435) with a mean of 2235539 kg/yr based on 5 years of trade data |
| Rice | Pert(326799,444027.4,743585) with a mean of 474415.6 kg/yr based on 5 years of trade data |
| Root veg | Pert(1614,3929.5,6517) with a mean of 3974.8 kg/yr based on last 5 years of trade data |
| Saltwater fish | Pert(48725,122948.8,205396) with a mean of 122948.8 kg/yr based on last 5 years of trade data |
| Seafood other | Pert(50,36479,99270) with a mean of 40872.69 kg/yr based on last 13 years of trade data due to high variability between years |
| Seaweed and algae | Pert(5650,12116.7,18451) with a mean of 12094.6 kg/yr based on 5 years of trade data |
| Shoots | Pert(15570,24689.9,44243) with a mean of 26428.8 kg/yr based on 5 years trade data |
| Soft beverages | Pert(519248,1626389,5340287) with a mean of 2060849 kg/yr based on 5 years of trade data |

Table A2: Weight of food consumed per UK consumer, by food and by age (kg/yr)

Assumptions made for restricted foods are shown in Table A3. Distributions were determined by Akaike’s Information Criterion in @Risk.

| **Food group** | **Age Category** | **Distribution and values (mean, 97.5^th^ percentile) kg/yr** |
| --- | --- | --- |
| Alcoholic beverages | Infant (4 - 18 months) | 0 |
| Alcoholic beverages | Child 1 (18 months - <5 years) | 0 |
| Alcoholic beverages | Child 2 (5 - <10 years) | 0 |
| Alcoholic beverages | Child 3 (10 - <16 years) | 0 |
| Alcoholic beverages | Adults (16 - <70 years) | Lognormal (102.29, 468.76) |
| Alcoholic beverages | Adults (>70 years) | Lognormal (102.29, 468.76) |
| Alcoholic beverages | Women of childbearing age (16-<50 years) | Lognormal (71.62, 286.16) |
| Baby food | Infant (4 - 18 months) | Lognormal (41.069, 165.689) |
| Baby food | Child 1 (18 months - <5 years) | Lognormal (16.13, 94.703) |
| Baby food | Child 2 (5 - <10 years) | Lognormal (7.283, 48.066) |
| Bread | Infant (4 - 18 months) | Lognormal (8.772, 25.608) |
| Bread | Child 1 (18 months - <5 years) | Lognormal (18.012, 42.472) |
| Bread | Child 2 (5 - <10 years) | Lognormal (25.637, 57.586) |
| Bread | Child 3 (10 - <16 years) | Lognormal (29.561, 71.412) |
| Bread | Adults (16 - <70 years) | Lognormal (31.436, 74.802) |
| Bread | Adults (>70 years) | Lognormal (31.436, 74.802) |
| Bread | Women of childbearing age (16-<50 years) | Lognormal (26.314, 58.211) |
| Cattle | Infant (4 - 18 months) | Lognormal (3.598, 12.901) |
| Cattle | Child 1 (18 months - <5 years) | Lognormal (5.087, 15.805) |
| Cattle | Child 2 (5 - <10 years) | Lognormal (7.121, 21.384) |
| Cattle | Child 3 (10 - <16 years) | Lognormal (9.652, 25.465) |
| Cattle | Adults (16 - <70 years) | Lognormal (13.173, 37.55) |
| Cattle | Adults (>70 years) | Lognormal (13.173, 37.55) |
| Cattle | Women of childbearing age (16-<50 years) | Lognormal (10.911, 30.83) |
| Cereals and grains | Infant (4 - 18 months) | Lognormal (2.207, 8.964) |
| Cereals and grains | Child 1 (18 months - <5 years) | Lognormal (2.842, 9.886) |
| Cereals and grains | Child 2 (5 - <10 years) | Lognormal (3.661, 12.872) |
| Cereals and grains | Child 3 (10 - <16 years) | Lognormal (4, 14.501) |
| Cereals and grains | Adults (16 - <70 years) | Lognormal (5.132, 20.975) |
| Cereals and grains | Adults (>70 years) | Lognormal (5.132, 20.975) |
| Cereals and grains | Women of childbearing age (16-<50 years) | Lognormal (4.203, 17.695) |
| Condiments, spices, preserves | Infant (4 - 18 months) | Lognormal (0.618, 2.921) |
| Condiments, spices and preserves | Child 1 (18 months - <5 years) | Lognormal (1.534, 6.44) |
| Condiments, spices and preserves | Child 2 (5 - <10 years) | Lognormal (1.914, 7.406) |
| Condiments, spices and preserves | Child 3 (10 - <16 years) | Lognormal (1.786, 7.636) |
| Condiments, spices and preserves | Adults (16 - <70 years) | Lognormal (2.6, 11.709) |
| Condiments, spices and preserves | Adults (>70 years) | Lognormal (2.6, 11.709) |
| Condiments, spices and preserves | Women of childbearing age (16-<50 years) | Lognormal (2.284, 10.613) |
| Confectionary | Infant (4 - 18 months) | Lognormal (10.022, 39.745) |
| Confectionary | Child 1 (18 months - <5 years) | Lognormal (20.896, 62.365) |
| Confectionary | Child 2 (5 - <10 years) | Lognormal (34.68, 83.865) |
| Confectionary | Child 3 (10 - <16 years) | Lognormal (34.834, 90.137) |
| Confectionary | Adults (16 - <70 years) | Lognormal (25.19, 78.502) |
| Confectionary | Adults (>70 years) | Lognormal (25.19, 78.502) |
| Confectionary | Women of childbearing age (16-<50 years) | Lognormal (23.533, 72.835) |
| Dairy products | Infant (4 - 18 months) | Lognormal (76.913, 268.241) |
| Dairy products | Child 1 (18 months - <5 years) | Lognormal (114.461, 292.153) |
| Dairy products | Child 2 (5 - <10 years) | Lognormal (92.425, 234.554) |
| Dairy products | Child 3 (10 - <16 years) | Lognormal (79.404, 230.201) |
| Dairy products | Adults (16 - <70 years) | Lognormal (80.495, 216.485) |
| Dairy products | Adults (>70 years) | Lognormal (80.495, 216.485) |
| Dairy products | Women of childbearing age (16-<50 years) | Lognormal (68.898, 182.416) |
| Dried fish | Infant (4 - 18 months) | Lognormal (1.978, 1.978) |
| Dried fish | Child 1 (18 months - <5 years) | Lognormal (3.915, 3.915) |
| Dried fish | Child 2 (5 - <10 years) | Lognormal (0.046, 0.046) |
| Dried fish | Child 3 (10 - <16 years) | Lognormal (0.289, 0.289) |
| Dried fish | Adults (16 - <70 years) | Lognormal (4.492, 17.99) |
| Dried fish | Adults (>70 years) | Lognormal (4.492, 17.99) |
| Dried fish | Women of childbearing age (16-<50 years) | Lognormal (4.492, 17.99) |
| Dried food, nuts and seeds | Infant (4 - 18 months) | Lognormal (1.418, 7.384) |
| Dried food, nuts and seeds | Child 1 (18 months - <5 years) | Lognormal (2.261, 11.827) |
| Dried food, nuts and seeds | Child 2 (5 - <10 years) | Lognormal (1.697, 8.995) |
| Dried food, nuts and seeds | Child 3 (10 - <16 years) | Lognormal (1.487, 8.744) |
| Dried food, nuts and seeds | Adults (16 - <70 years) | Lognormal (3.218, 19.041) |
| Dried food, nuts and seeds | Adults (>70 years) | Lognormal (3.218, 19.041) |
| Dried food, nuts and seeds | Women of childbearing age (16-<50 years) | Lognormal (2.59, 14.425) |
| Eggs | Infant (4 - 18 months) | Lognormal (2.99, 13.69) |
| Eggs | Child 1 (18 months - <5 years) | Lognormal (4.055, 16.451) |
| Eggs | Child 2 (5 - <10 years) | Lognormal (4.528, 19.423) |
| Eggs | Child 3 (10 - <16 years) | Lognormal (5.149, 21.554) |
| Eggs | Adults (16 - <70 years) | Lognormal (8.197, 31.218) |
| Eggs | Adults (>70 years) | Lognormal (8.197, 31.218) |
| Eggs | Women of childbearing age (16-<50 years) | Lognormal (7.197, 31.208) |
| Fats and oils | Infant (4 - 18 months) | Lognormal (1.874, 6.284) |
| Fats and oils | Child 1 (18 months - <5 years) | Lognormal (4.987, 10.553) |
| Fats and oils | Child 2 (5 - <10 years) | Lognormal (7.496, 14.617) |
| Fats and oils | Child 3 (10 - <16 years) | Lognormal (8.693, 19.052) |
| Fats and oils | Adults (16 - <70 years) | Lognormal (8.047, 18.867 ) |
| Fats and oils | Adults (>70 years) | Lognormal (8.047, 18.867 ) |
| Fats and oils | Women of childbearing age (16-<50 years) | Lognormal (7.311, 18.205) |
| Freshwater fish | Infant (4 - 18 months) | Lognormal (10.038, 10.038) |
| Freshwater fish | Child 1 (18 months - <5 years) | Lognormal (10.038, 10.038) |
| Freshwater fish | Child 2 (5 - <10 years) | Lognormal (8.092, 10.142) |
| Freshwater fish | Child 3 (10 - <16 years) | Lognormal (7.711, 13.078) |
| Freshwater fish | Adults (16 - <70 years) | Lognormal (10.167, 28.668) |
| Freshwater fish | Adults (>70 years) | Lognormal (10.167, 28.668) |
| Freshwater fish | Women of childbearing age (16-<50 years) | Lognormal (9.982, 10.95) |
| Fruit | Infant (4 - 18 months) | Lognormal (18.468, 56.073) |
| Fruit | Child 1 (18 months - <5 years) | Lognormal (33.891, 105.194) |
| Fruit | Child 2 (5 - <10 years) | Lognormal (39.242, 115.428) |
| Fruit | Child 3 (10 - <16 years) | Lognormal (33.991, 116.234) |
| Fruit | Adults (16 - <70 years) | Lognormal (27.347, 92.487) |
| Fruit | Adults (>70 years) | Lognormal (27.347, 92.487) |
| Fruit | Women of childbearing age (16-<50 years) | Lognormal (24.877, 83.319) |
| Infant formula | Infant (4 - 18 months) | Lognormal (160.094, 322) |
| Infant formula | Child 1 (18 months - <5 years) | 0 |
| Infant formula | Child 2 (5 - <10 years) | 0 |
| Infant formula | Child 3 (10 - <16 years) | 0 |
| Infant formula | Adults (16 - <70 years) | 0 |
| Infant formula | Adults (>70 years) | 0 |
| Infant formula | Women of childbearing age (16-<50 years) | 0 |
| Leguminous vegetables | Infant (4 - 18 months) | Lognormal (4.333, 17.15) |
| Leguminous vegetables | Child 1 (18 months - <5 years) | Lognormal (5.796, 19.103) |
| Leguminous vegetables | Child 2 (5 - <10 years) | Lognormal (7.934, 25.239) |
| Leguminous vegetables | Child 3 (10 - <16 years) | Lognormal (7.591, 26.998) |
| Leguminous vegetables | Adults (16 - <70 years) | Lognormal (11.311, 40.627) |
| Leguminous vegetables | Adults (>70 years) | Lognormal (11.311, 40.627) |
| Leguminous vegetables | Women of childbearing age (16-<50 years) | Lognormal (10.08, 36.085) |
| Meat and dairy alternatives | Infant (4 - 18 months) | Lognormal (9.94, 68.424) |
| Meat and dairy alternatives | Child 1 (18 months - <5 years) | Lognormal (19.227,142.504) |
| Meat and dairy alternatives | Child 2 (5 - <10 years) | Lognormal (11.86, 65.354) |
| Meat and dairy alternatives | Child 3 (10 - <16 years) | Lognormal (10.151,63.012) |
| Meat and dairy alternatives | Adults (16 - <70 years) | Lognormal (21.681, 104.667) |
| Meat and dairy alternatives | Adults (>70 years) | Lognormal (21.681, 104.667) |
| Meat and dairy alternatives | Women of childbearing age (16-<50 years) | Lognormal (18.77, 100.387) |
| Mushrooms | Infant (4 - 18 months) | Lognormal (1.1, 5.271 |
| Mushrooms | Child 1 (18 months - <5 years) | Lognormal (0.98,3.987) |
| Mushrooms | Child 2 (5 - <10 years) | Lognormal (1.332, 6.724) |
| Mushrooms | Child 3 (10 - <16 years) | Lognormal (2.03,8.982) |
| Mushrooms | Adults (16 - <70 years) | Lognormal (4.729,18.55) |
| Mushrooms | Adults (>70 years) | Lognormal (4.729,18.55) |
| Mushrooms | Women of childbearing age (16-<50 years) | Lognormal (4.082, 18.055) |
| Non leguminous green vegetables | Infant (4 - 18 months) | Lognormal (5.324, 22.903) |
| Non leguminous green vegetables | Child 1 (18 months - <5 years) | Lognormal (6.892, 22.285) |
| Non leguminous green vegetables | Child 2 (5 - <10 years) | Lognormal (9.296, 32.613) |
| Non leguminous green vegetables | Child 3 (10 - <16 years) | Lognormal (9.836, 32.055) |
| Non leguminous green vegetables | Adults (16 - <70 years) | Lognormal (16.316, 53.692) |
| Non leguminous green vegetables | Adults (>70 years) | Lognormal (16.316, 53.692) |
| Non leguminous green vegetables | Women of childbearing age (16-<50 years) | Lognormal (15.603, 50.136) |
| Other meats | Infant (4 - 18 months) | Lognormal (5.035, 17.435) |
| Other meats | Child 1 (18 months - <5 years) | Lognormal (8.842, 24.227) |
| Other meats | Child 2 (5 - <10 years) | Lognormal (12.991, 33.204) |
| Other meats | Child 3 (10 - <16 years) | Lognormal (16.197, 43.97) |
| Other meats | Adults (16 - <70 years) | Lognormal (20.269, 56.565) |
| Other meats | Adults (>70 years) | Lognormal (20.269, 56.565) |
| Other meats | Women of childbearing age (16-<50 years) | Lognormal (17.507, 47.829) |
| Pasta | Infant (4 - 18 months) | Lognormal (6.354, 21.928) |
| Pasta | Child 1 (18 months - <5 years) | Lognormal (9.349, 27.681) |
| Pasta | Child 2 (5 - <10 years) | Lognormal (13.043, 38.962) |
| Pasta | Child 3 (10 - <16 years) | Lognormal (17.318, 46.929) |
| Pasta | Adults (16 - <70 years) | Lognormal (19.797, 63.665) |
| Pasta | Adults (>70 years) | Lognormal (19.797, 63.665) |
| Pasta | Women of childbearing age (16-<50 years) | Lognormal (19.277, 54.659) |
| Potatoes | Infant (4 - 18 months) | Lognormal (12.445, 40.973) |
| Potatoes | Child 1 (18 months - <5 years) | Lognormal (16.3, 45.168) |
| Potatoes | Child 2 (5 - <10 years) | Lognormal (25.368, 61.289) |
| Potatoes | Child 3 (10 - <16 years) | Lognormal (30.631, 74.713) |
| Potatoes | Adults (16 - <70 years) | Lognormal (34.115, 87.076) |
| Potatoes | Adults (>70 years) | Lognormal (34.115, 87.076) |
| Potatoes | Women of childbearing age (16-<50 years) | Lognormal (29.541, 78.084) |
| Ready-to-eat foods | Infant (4 - 18 months) | Lognormal (11.524, 53.762) |
| Ready-to-eat foods | Child 1 (18 months - <5 years) | Lognormal (18.451, 59.687) |
| Ready-to-eat foods | Child 2 (5 - <10 years) | Lognormal (24.614, 75.624) |
| Ready-to-eat foods | Child 3 (10 - <16 years) | Lognormal (33.171, 97.221) |
| Ready-to-eat foods | Adults (16 - <70 years) | Lognormal (35.473, 118.625) |
| Ready-to-eat foods | Adults (>70 years) | Lognormal (35.473, 118.625) |
| Ready-to-eat foods | Women of childbearing age (16-<50 years) | Lognormal (32.307, 110.834) |
| Rice | Infant (4 - 18 months) | Lognormal (5.351, 26.183) |
| Rice | Child 1 (18 months - <5 years) | Lognormal (6.308, 30.458) |
| Rice | Child 2 (5 - <10 years) | Lognormal (9.088, 42.372) |
| Rice | Child 3 (10 - <16 years) | Lognormal (12.512, 52.887) |
| Rice | Adults (16 - <70 years) | Lognormal (19.44, 79.875) |
| Rice | Adults (>70 years) | Lognormal (19.44, 79.875) |
| Rice | Women of childbearing age (16-<50 years) | Lognormal (18.474, 75.21) |
| Root vegetables | Infant (4 - 18 months) | Lognormal (10.084, 34.216) |
| Root vegetables | Child 1 (18 months - <5 years) | Lognormal (5.658, 21.951) |
| Root vegetables | Child 2 (5 - <10 years) | Lognormal (7.655, 28.22) |
| Root vegetables | Child 3 (10 - <16 years) | Lognormal (8.098, 29.297) |
| Root vegetables | Adults (16 - <70 years) | Lognormal (15.084, 52.191) |
| Root vegetables | Adults (>70 years) | Lognormal (15.084, 52.191) |
| Root vegetables | Women of childbearing age (16-<50 years) | Lognormal (13.821, 47.477) |
| Saltwater fish | Infant (4 - 18 months) | Lognormal (3.947, 12.507) |
| Saltwater fish | Child 1 (18 months - <5 years) | Lognormal (5.569, 15.972) |
| Saltwater fish | Child 2 (5 - <10 years) | Lognormal (6.872, 20.973) |
| Saltwater fish | Child 3 (10 - <16 years) | Lognormal (8.503, 26.08) |
| Saltwater fish | Adults (16 - <70 years) | Lognormal (13.253, 40.599) |
| Saltwater fish | Adults (>70 years) | Lognormal (13.253, 40.599) |
| Saltwater fish | Women of childbearing age (16-<50 years) | Lognormal (11.009, 33.709) |
| Seafood other | Infant (4 - 18 months) | Lognormal (1.229, 5.533) |
| Seafood other | Child 1 (18 months - <5 years) | Lognormal (1.54, 4.822) |
| Seafood other | Child 2 (5 - <10 years) | Lognormal (2.686, 9.82) |
| Seafood other | Child 3 (10 - <16 years) | Lognormal (3.041, 10.69) |
| Seafood other | Adults (16 - <70 years) | Lognormal (6.289, 24.552) |
| Seafood other | Adults (>70 years) | Lognormal (6.289, 24.552) |
| Seafood other | Women of childbearing age (16-<50 years) | Lognormal (6.117, 26.521) |
| Seaweed and algae | Infant (4 - 18 months) | Lognormal (0.104, 0.32) |
| Seaweed and algae | Child 1 (18 months - <5 years) | Lognormal (0.208, 0.474) |
| Seaweed and algae | Child 2 (5 - <10 years) | Lognormal (0.208, 0.474) |
| Seaweed and algae | Child 3 (10 - <16 years) | Lognormal (0.313, 0.856) |
| Seaweed and algae | Adults (16 - <70 years) | Lognormal (0.368, 1.366) |
| Seaweed and algae | Adults (>70 years) | Lognormal (0.368, 1.366) |
| Seaweed and algae | Women of childbearing age (16-<50 years) | Lognormal (0.397, 1.387) |
| Shoots | Infant (4 - 18 months) | Lognormal (0.00043, 0.071, 0.566) fitted to 2.5th, mean and 99.9th percentile |
| Shoots | Child 1 (18 months - <5 years) | Lognormal (0.000464, 0.05, 0.739) fitted to 2.5th, mean and 99.9th percentile |
| Shoots | Child 2 (5 - <10 years) | Lognormal (0.001717, 0.068, 0.181) fitted to 2.5th, mean and 97.5th percentile |
| Shoots | Child 3 (10 - <16 years) | Lognormal (0.000602, 0.181, 1.179) fitted to 2.5th, mean and 97.5th percentile |
| Shoots | Adults (16 - <70 years) | Lognormal (0.000803, 0.313, 6.378) fitted to 2.5th, mean and 99.9th percentile |
| Shoots | Adults (>70 years) | Lognormal (0.000803, 0.313, 6.378) fitted to 2.5th, mean and 99.9th percentile |
| Shoots | Women of childbearing age (16-<50 years) | Lognormal (0.000783, 0.236, 1.576) fitted to 2.5th, mean and 97.5th percentile |
| Soft beverages | Infant (4 - 18 months) | Lognormal (0.41, 0.558) |
| Soft beverages | Child 1 (18 months - <5 years) | Lognormal (0.41, 0.558) |
| Soft beverages | Child 2 (5 - <10 years) | Lognormal (1.246, 3.763) |
| Soft beverages | Child 3 (10 - <16 years) | Lognormal (12.24, 44.88) |
| Soft beverages | Adults (16 - <70 years) | Lognormal (58.911, 217.944) |
| Soft beverages | Adults (>70 years) | Lognormal (58.911, 217.944) |
| Soft beverages | Women of childbearing age (16-<50 years) | Lognormal (55.09, 199.508) |

Table A3: Exceptions and assumptions for certain food groups

| **Food group** | **Exceptions** | **Assumption** |
| --- | --- | --- |
| Alcohol beverages | Only Adult consumption data used | The supply of alcohol to under 18’s is not legally permitted |
| Baby food | Only Infant, Child 1 and Child 2 data used | Only Infant, Child 1 and Child 2 consumption data available |
| Infant formula | Only Infant consumption data used | Infant formula is only appropriate for infants |
| Freshwater fish | Child 1 consumption data used for infant | No infant consumption data |
| Dried fish | Adult consumption data used for women of childbearing age | No consumption data for women of childbearing age (to represent foetal exposure) |
| Seaweed and algae | Child 2 consumption data used for Child 1 | No Child 1 consumption data |
| Soft beverages | Child 1 consumption data used for infant | No infant consumption data |

Table A4. Percentage of population in UK by age category (ONS, 2019)

| $\boldsymbol{a}$ ***(age group)*** | ***P_age*** |
| --- | --- |
| Infant (4 - 18 months) | 0.016 |
| Child 1 (18 months - <5 years) | 0.041 |
| Child 2 (5 - <10 years) | 0.062 |
| Child 3 (10 - <16 years) | 0.070 |
| Adults (16 - <70 years) excluding females (16-<50 years) | 0.460 |
| Female (16-<50 years) | 0.215 |
| Over 70 | 0.135 |

Table A5: Activity concentration distributions for each commodity, based on sample data provided in the MHLW monitoring data.

| **Food group** | **Distribution and values** |
| --- | --- |
| Alcoholic beverages | Weibull(1.3699,5.8112,RiskShift(0.76593)) truncated at 0 |
| Baby Food | ExtvalueMin(8.306,1.1167) Exponential family, truncated at 0 |
| Bread | Extvalue(7.993,2.1749) Exponential family, truncated at 0 |
| Cattle | Expon(23.178,Shift(1.4)) Exponential family, truncated at 0 |
| Cereals and grains | Cumulative distribution fitted to minimum and percentile observed data. Maximum value was +25% higher than observed maximum value. |
| Condiments, spices, preserves | Extvalue(9.1268,4.7069) Exponential family truncated at 0 |
| Confectionary | Weibull(2.0003,8.5278,Shift(2.3871)) truncated at 0 |
| Dairy products | Gamma(3.0282,1.6115,Shift(-0.1451)) truncated at 0 |
| Dried fish | Logistic(14.6082,3.3915) truncated at 0 |
| Dried food, nuts and seeds | Loglogistic(0.72596,11.331,2.6351) truncated at 0 |
| Eggs | Logistic(15.7881,2.8469) truncated at 0 |
| Fats and oils | Pearson5(9.0089,55.501,Shift(0.52163)) Inverse Gamma truncated at 0 |
| Freshwater fish | Cumulative distribution fitted to minimum and percentile observed data. Maximum value was +25% higher than observed maximum value. |
| Fruit | Cumulative distribution fitted to minimum and percentile observed data. Maximum value was +25% higher than observed maximum value. |
| Infant formula | ExtvalueMin(7.8747,1.826) Exponential family truncated at 0 |
| Leguminous green vegetables | Loglogistic (0.83269,8.4946,3.1578) truncated at 0 |
| Meat and dairy alternatives | ExtvalueMin(14.2062,6.7596) Exponential family truncated at 0 |
| Mushrooms | Cumulative distribution fitted to minimum and percentile observed data. Maximum value was +25% higher than observed maximum value. |
| NLGV | Cumulative distribution fitted to minimum and percentile observed data. Maximum value was +25% higher than observed maximum value. |
| **Food group** | **Distribution and values** |
| Other meats | ExtvalueMin(19.2791,5.7746) Exponential family truncated at 0 |
| Pasta | Weibull(1.9917,10.33,Shift(1.5646)) truncated at 0 |
| Potatoes | Logistic(9.8555,3.3932) truncated at 0 |
| RTE foods | Lognorm(11.551,5.3608,Shift(-0.59879)) truncated at 0 |
| Rice | Cumulative distribution fitted to minimum and percentile observed data. Maximum value was +25% higher than observed maximum value. |
| Root veg | Pearson5(7.3849,102.37,Shift(-4.5292)) Inverse gamma truncated at 0 |
| Saltwater fish | Cumulative distribution fitted to minimum and percentile observed data. Maximum value was +25% higher than observed maximum value. |
| Seafood other | Cumulative distribution fitted to minimum and percentile observed data. Maximum value was +25% higher than observed maximum value. |
| Seaweed and algae | Logistic(19.1265,2.3681) truncated at 0 |
| Shoots | Lognorm(21.454,24.84,Shift(0.97131)) truncated at 0 |
| Soft beverages | Invgauss(3.0018,1.7885,Shift(0.23436)) truncated at 0 |

Table A6: ICRP Dose coefficients by age group (mSv/Bq)

| **Age group** | **Dose coefficient Cs-134** | **Dose coefficient Cs-137** |
| --- | --- | --- |
| Infant (4 - 18 months) | 1.6 x 10^-5^ | 1.2 x 10^-5^ |
| Child 1 (18 months - <5 years) | 1.6 x 10^-5^ | 1.2 x 10^-5^ |
| Child 2 (5 - <10 years) | 1.4 x 10^-5^ | 1.0 x 10^-5^ |
| Child 3 (10 - <16 years) | 1.9 x 10^-5^ | 1.3 x 10^-5^ |
| Adults (16 - <70 years) / Females (16-<50) | 1.9 x 10^-5^ | 1.3 x 10^-5^ |
| Woman of childbearing age (16-<50) (foetus) | 8.7 x 10^-5^ | 5.7 x 10^-5^ |

Table A7: Estimated annual activity ingested (Bq per yr)

for a representative UK consumer by age.

| Age | $\boldsymbol{N\_nocontrols}_{\boldsymbol{age}}$  Mean (5^th^, 95^th^)  Bq/yr | $\boldsymbol{N\_controls}_{\boldsymbol{age}}$  Mean (5^th^, 95^th^)  Bq/yr | $\boldsymbol{N\_difference}_{\boldsymbol{age}}$  Mean (5^th^, 95^th^)  Bq/yr |
| --- | --- | --- | --- |
| Infant (4 – 18 months) | 34.4 (0.5-138.7) | 34.3 (0.5, 138.5) | 0.14 (0, 3.6x10^-15^) |
| Child 1 (18 months - <5 years) | 57.6 (1.6, 269.2) | 57.7 (1.6, 269.4) | 0.15 (0, 7.1 x10^-15)^ |
| Child 2 (5 - <10 years) | 69.5 (2.3, 336.8) | 69.9 (2.3, 336.5) | 0.18 (0, 7.11x10^-15^) |
| Child 3 (10 - <16 years) | 75.4 (1.7, 393.6) | 73.3 (1.7, 393.9) | 0.42 (0, 7.11x10^-15^) |
| Adults (16 - <70 years) excluding females (16-<50 years) | 100.6 (2.2, 452.1) | 99.8 (2.2, 452.03) | 0.5 (0,1.2 x10^-14^) |
| Female (16-<50 years) | 92.4 (1.8, 439.5) | 91.6 (1.8, 438.2) | 0.43(0, 8.44 x10^-15^) |
